# Comparative transcriptome analysis of rice cultivars resistant and susceptible to *Rhizoctonia solani* AG1-IA

**DOI:** 10.1186/s12864-022-08816-x

**Published:** 2022-08-19

**Authors:** Yan Wang, Hang Luo, Haining Wang, Zongjing Xiang, Songhong Wei, Wenjing Zheng

**Affiliations:** 1 College of Plant Protection, Department of Plant Pathology, Shenyang Agricultural University, Shenyang, 110866 Liaoning China; 2 Liaoning Rice Research Institute, Shenyang, 110101 Liaoning China

**Keywords:** Rice sheath blight, Transcriptome, Resistance mechnism, Phenylalanine metabolism

## Abstract

**Background:**

Rice sheath blight, which is caused by *Rhizoctonia solani*, is the most destructive disease affecting rice production, but the resistance mechanism to this pathogen has not been fully elucidated.

**Results:**

In this study, we selected two rice cultivars based on their resistance to the pathogen and analyzed and compared the transcriptomic profiles of two cultivars, the moderately resistant variety Gangyuan8 and the highly susceptible variety Yanfeng47, at different time points after inoculation. The comparative transcriptome profiling showed that the expression of related genes gradually increased after pathogen inoculation. The number of differentially expressed genes (DEGs) in Yanfeng47 was higher than that in Gangyuan8, and this result revealed that Yanfeng47 was more susceptible to fungal attack. At the early stage (24 and 48 h), the accumulation of resistance genes and a resistance metabolism occurred earlier in Ganguan8 than in Yanfeng47, and the resistance enrichment entries were more abundant in Ganguan8 than in Yanfeng47.

**Conclusions:**

Based on the GO and KEGG enrichment analyses at five infection stages, we concluded that phenylalanine metabolism and the jasmonic acid pathway play a crucial role in the resistance of rice to sheath blight. Through a comparative transcriptome analysis, we preliminarily analyzed the molecular mechanism responsible for resistance to sheath blight in rice, and the results lay the foundation for the development of gene mining and functional research on rice resistance to sheath blight.

**Supplementary Information:**

The online version contains supplementary material available at 10.1186/s12864-022-08816-x.

## Background

Rice sheath blight (RHB) is one of the three major rice diseases that seriously affects the yield and quality of rice and was first discovered by Japanese researchers in 1910 [15, 38, 44]. The planting of resistant varieties is considered the most effective method for controlling the disease [40, 50]. To date, varieties with high resistance or immunity that carry major genes have not been identified [54]. To date, many QTLs for rice resistance to sheath blight have been reported, and pyramiding multiple QTLs has proven to be a feasible strategy for improving rice resistance to disease [5, 7, 29, 32, 55]. *Rhizoctonia solani*, a soil-borne Basidiomycete fungus, can be divided into 14 groups, and among these groups, AG1 is the main hyphal fusion group and the major pathogenic bacterium responsible for rice sheath blight [38, 52].

With the development of sequencing technology, next-generation sequencing technology is characterized by high throughput and low cost [18] and has opened up new research directions involving the study of gene structure, expression and function and greatly promoted omics analyses [45]. The physiological and biochemical reactions and phenotypic changes induced by plants in response to pathogen infection are determined by related genes [12, 35]. These genes participate in multiple molecular regulation pathways and cross each other to form a set of complex disease resistance regulation networks to ultimately achieve disease resistance [30, 44]. Due to their complexity, rice disease resistance mechanisms are difficult to analyze using traditional methods. The defense mode, response and pathogenic mechanism during the interaction between rice and fungi are very complex [51]. Although many studies have been performed, no breakthroughs have been achieved, and research on rice sheath blight still faces many difficulties [39]. In this study, RNA-seq technology was used to conduct a comparative transcriptome analysis of infected leaves at the tillering stage. Gangyuan8 (GG), a moderately resistant variety, and Yanfeng47 (YY), a susceptible variety, were analyzed at 0, 24, 48, 72 and 96 h after AG1 IA inoculation. A transcriptome analysis was performed to explore the resistance mechanism to rice sheath blight. Bioinformatics and qRT-PCR analyses were performed to investigate how two rice cultivars induce resistance or susceptibility to *R. solani* and to identify the metabolic pathways associated with this response. To our knowledge, this study involves the first use of production varieties for a comparative transcriptome profiling analysis of the response to AG1 IA infection.

## Results

### Selection and determination of sampling time points

To determine the time points for transcriptome sampling, we used fugus plate cultured with *R. solani* AG1 IA for 2 days, inoculated the middle of the leaves of the different varieties at the tillering stage and wrapped the leaves with plastic wrap. The results showed that 24 h after inoculation, mycelia were attached to the leaf surface of Yanfeng47. In addition, 48 h after inoculation, disease spots began to form on the leaf surface of Yanfeng47, and Gangyuan8 also started to show disease spots. Seventy-two hours after inoculation, the lesion area of Yanfeng47 had expanded, and Gangyuan8 also showed obvious lesion. Ninety-six hours after inoculation, the leaves of Yanfeng47 had wilted, and the lesion area of Gangyuan8 was larger. Consistent with the results of previous studies, the resistance of Gangyuan8 and Yanfeng47 to *R. solani* AG1 IA showed significant differences (Supplementary Figure S1 and Figure S2).

### RNA-seq results

To investigate the changes in the gene expression profiles of Gangyuan8 and Yanfeng47 leaves during the initial stage of AG1 infection, we performed a transcription analysis of leaves after AG1 IA inoculation by high-throughput sequencing. Transcriptome data were generated from the leaves at different time points. A transcriptome analysis of each variety was performed using 4 time points and 3 biological replicates of each time point. Thirty samples yielded 236.04 Gb of clean data, and 6.18 Gb of clean data were obtained from each sample. The Q30 base percentage was at least 93.91%. The clean reads from each sample were sequenced using the designated reference genome, and the alignment efficiency ranged from 76.72% to 95.72%. To examine the quality of the biological replicates, we calculated Pearson correlation coefficient (PCC) values for each pair of samples and performed a cluster analysis, which showed that the 96-h samples were clustered far from their replicated samples (Supplementary Figure S3). We also found that the 48- and 72-h samples were clustered together within the same species, which might indicate that the changes in gene expression within the samples from 48 to 72 h were not obvious. This result suggested that the clustering order of all the samples was roughly consistent with the sampling time. These samples were also subjected to a principal component analysis (PCA) (Supplementary Figure S4). The first and second principal components (PC1 and PC2) showed that the 96-h samples were separated from the other samples. The 0- and 24-h Yanfeng47 samples were also separated by a long distance.

### Differentially expressed genes

**Fig. 1:**
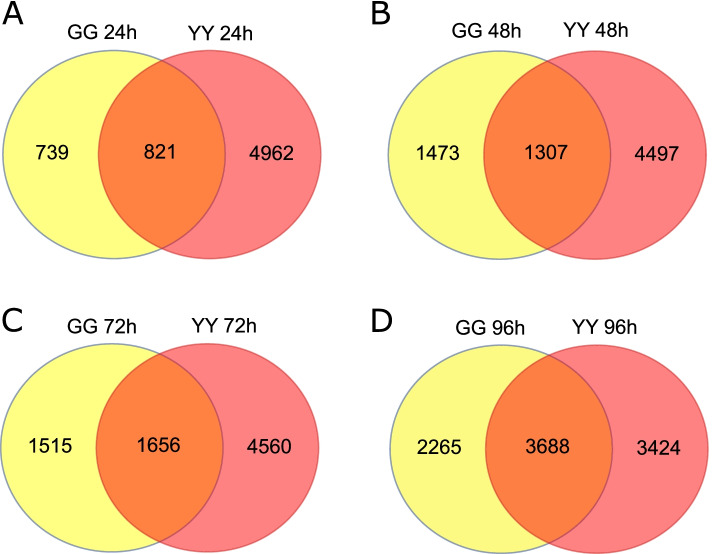
Venn diagram of differentially expressed genes found in both rice cultivars at the same time points after AG1 IA infection. GG 24 h, GG 48, GG72 and GG96 denote the differentially expressed gene sets obtained from Gangyuan8 leaves at 24, 48, 72 and 96 h after AG1 IA infection, respectively, and YY24 h, YY48 h, YY72 h and YY96 h denote the differentially expressed gene sets obtained from Yanfeng47 leaves at 24, 48, 72 and 96 h after AG1 IA infection

**Fig. 2:**
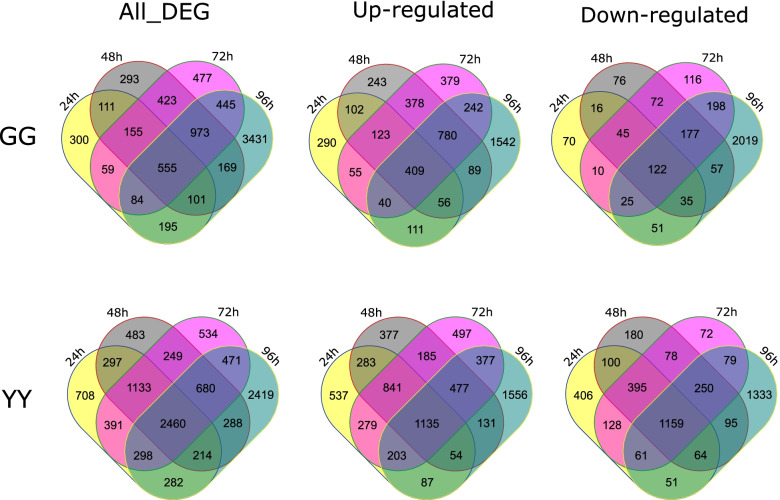
Venn diagram of differentially expressed genes in both rice cultivars at different time points after AG1 IA inoculation. GG24 h, GG48 h, GG72 h, and GG96 h denote the differentially expressed gene sets obtained by comparing Gangyuan8 samples at 24, 48, 72 and 96 h after AG1 IA inoculation, respectively, with the control sample at the same time point, and YY24 h, YY48 h, YY72 h and YY96 h denote the differentially expressed gene sets obtained by comparing the GG samples at 24, 48, 72 and 96 h after AG1 IA inoculation, respectively, with the control sample at the same time point. DEGs, differentially expressed genes; upregulated, upregulated DEGs; downregulated, downregulated DEGs

**Fig. 3:**
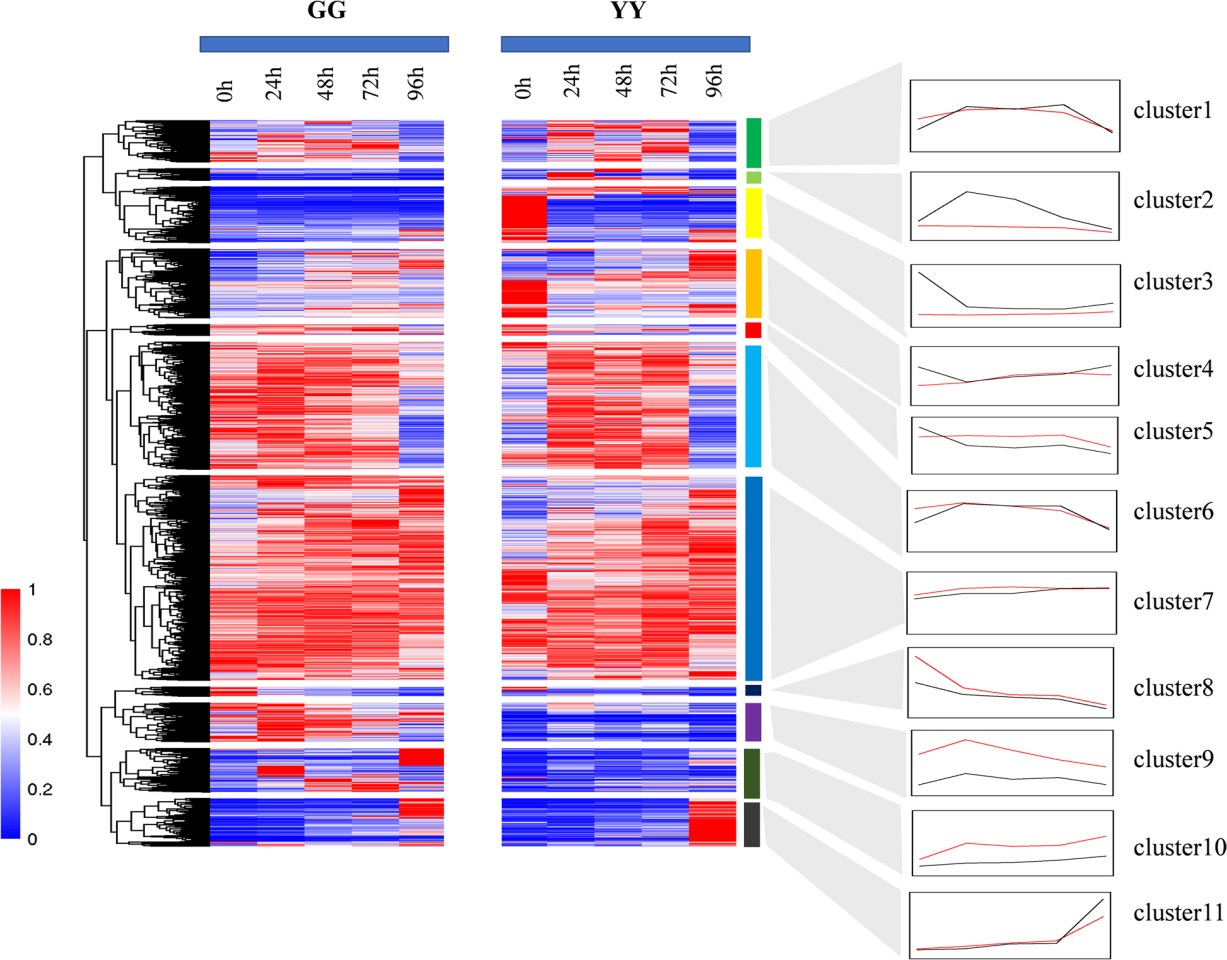
Temporal expression profiles of the 11 clusters of DEGs in the GG and YY leaves

**Fig. 4:**
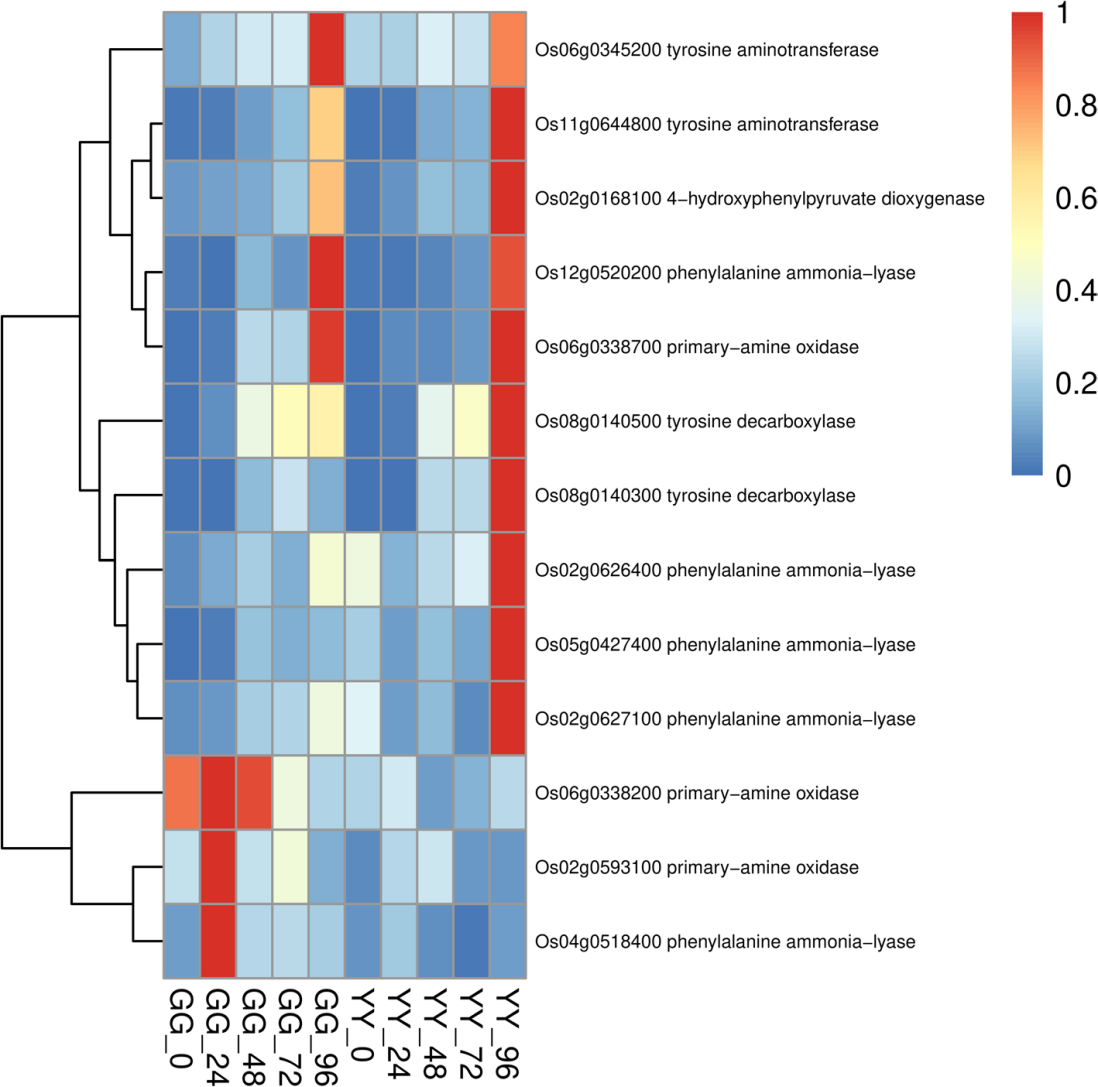
Detailed expression profiles of genes related to phenylalanine metabolism

Differentially expressed genes (DEGs) were identified by comparing the gene expression data obtained from Gangyuan8 and Yanfeng47 leaves at five time points. At all tested time points after inoculation, the number of upregulated genes exceeded the number of downregulated genes in both cultivars, and the number of upregulated genes in Yanfeng47 leaves was higher than that in Gangyuan8 leaves (Table S1). In both cultivars, the number of DEGs continued to increase from 0 to 96 h after inoculation. In Gangyuan8, the number of DEGs identified 96 h after inoculation was significantly higher than that found at other time points, and the number of DEGs showed an increasing trend from 0 to 96 h after inoculation. This study compared the DEGs of the two varieties at the same time points (GG24-YY24, GG48-YY48, GG72-YY72 and GG96-YY96; Fig. 1) and compared the DEGs of each variety at the different time points (GG24 h, GG48 h, GG72 h, GG96 h, YY24 h, YY48 h, YY72 h and YY96 h; Fig. 2). As shown in Fig. 3, 1656 and 3688 DEGs were identified in the two varieties at 72 and 96 h after inoculation, respectively, and the number of these DEGs was higher than that at other time points. In Gangyuan8 leaves, 555 DEGs were shared among the DEGs at 24, 48, 72 and 96 h after inoculation, and these included 409 upregulated genes and 122 downregulated genes. A total of 2460 DEGs were found in the Yanfeng47 leaves, and these included 1135 upregulated genes and 1159 downregulated genes. As indicated in Fig. 4, from 24 to 96 h after inoculation, the number of DEGs in Yanfeng47 leaves was higher than that in Gangyuan8 leaves.

### Functional enrichment analysis

The enriched DEGs between the control and inoculation treatments were classified by GO annotation, and we found that the enriched DEGs were involved in multiple biological activities (Figure S5). All unigenes were annotated in the GO database and classified into three main categories. Among biological processes, “chloroplast envelope”, “chloroplast stroma” and “plasma membrane” were the three processes showing the highest degree of gene enrichment. The analysis of molecular functions revealed that “iron ion binding” was the only process with the highest degree of enrichment. In the cellular component category, “pentose-phosphate shunt”, “response to cadmium ion”, “response to cold”, and “response to salt stress” were the four processes with the highest enrichment degree.

In this study, some GO pathways were enriched in only a few DEGs (Figure S6). GO: 0,016,168, GO: 0,018,298, GO: 0,016,023 and GO: 0,009,523 were enriched only in the GG_0_VS_24 and GG_0_VS_48 h comparisons. This result indicated that the functional pathways involving these genes may be related to the early response of the varieties to pathogens. Our results suggest that at early time points in the rice-AG1 IA interaction, the genes related to chlorophyll binding, protein-chromophore linkage, cytoplasmic membrane-bounded vesicle and photosystem II are more active in Gangyuan8 leaves than in Yanfeng47 leaves.

### Analysis of the gene expression profiles of rice cultivars

We also wanted to further analyze whether a certain gene is only expressed in the early stage of GG but not in YY; thus, these genes are likely related to the difference in the responses of the varieties to AG1 IA. Therefore, we extracted all DEGs and used them to generate expression profiles (Fig. 3). In the diagram, the red colors represent high expression, and the blue colors represent low expression. The GG sample is shown on the left, and the YY sample is presented on the right. A total of 11 clusters were obtained, and the expression information of each cluster is shown in the line chart on the right of Fig. 7. In this line chart, the red color represents the GG sample, and the black color represents the YY sample, as in cluster 9. The expression of these genes was high at 24 h and then decreased, and this profile is probably related to the early response of rice varieties to the pathogen. At the same time, the expression level of cluster 9 in the GG sample was higher than that in the YY sample, which may be due to the high expression of genes involved in resistance to infection in GG; thus, GG was identified as a disease-resistant variety. A similar trend was found for cluster 10. The analysis of clusters 9 and 10 obtained for GG revealed that the expression level of the genes related to cluster 9 increased continuously before 24 h and then decreased continuously after 24 h. In cluster 10, the increase and decreased occurred at 24 and 72 h, respectively. The GO enrichment results showed that the related genes in cluster 10 were mainly concentrated in the integrity of the membrane, metabolic process and cytoplasmic membrane-bound vesicle (Figure S7). The expression of cluster 11 in GG and YY remained highly after inoculation. In addition, the genes in cluster 11 were enriched in the response to salt stress, heme binding and extracellular matrix.

### Metabolic pathway analysis after AG1 IA inoculation

**Fig. 5:**
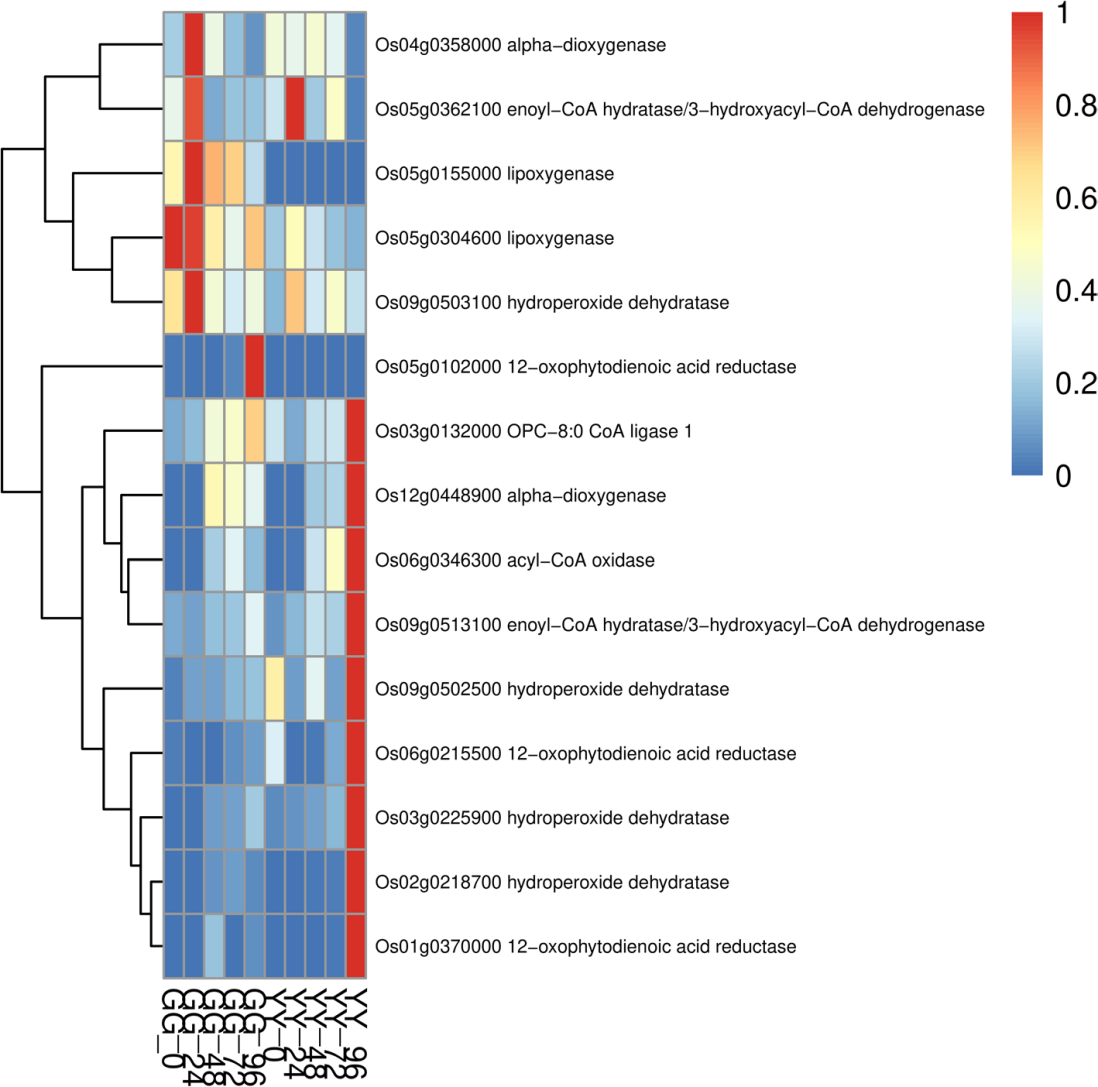
Detailed expression profiles of genes related to alpha-linolenic acid metabolism

**Fig. 6:**
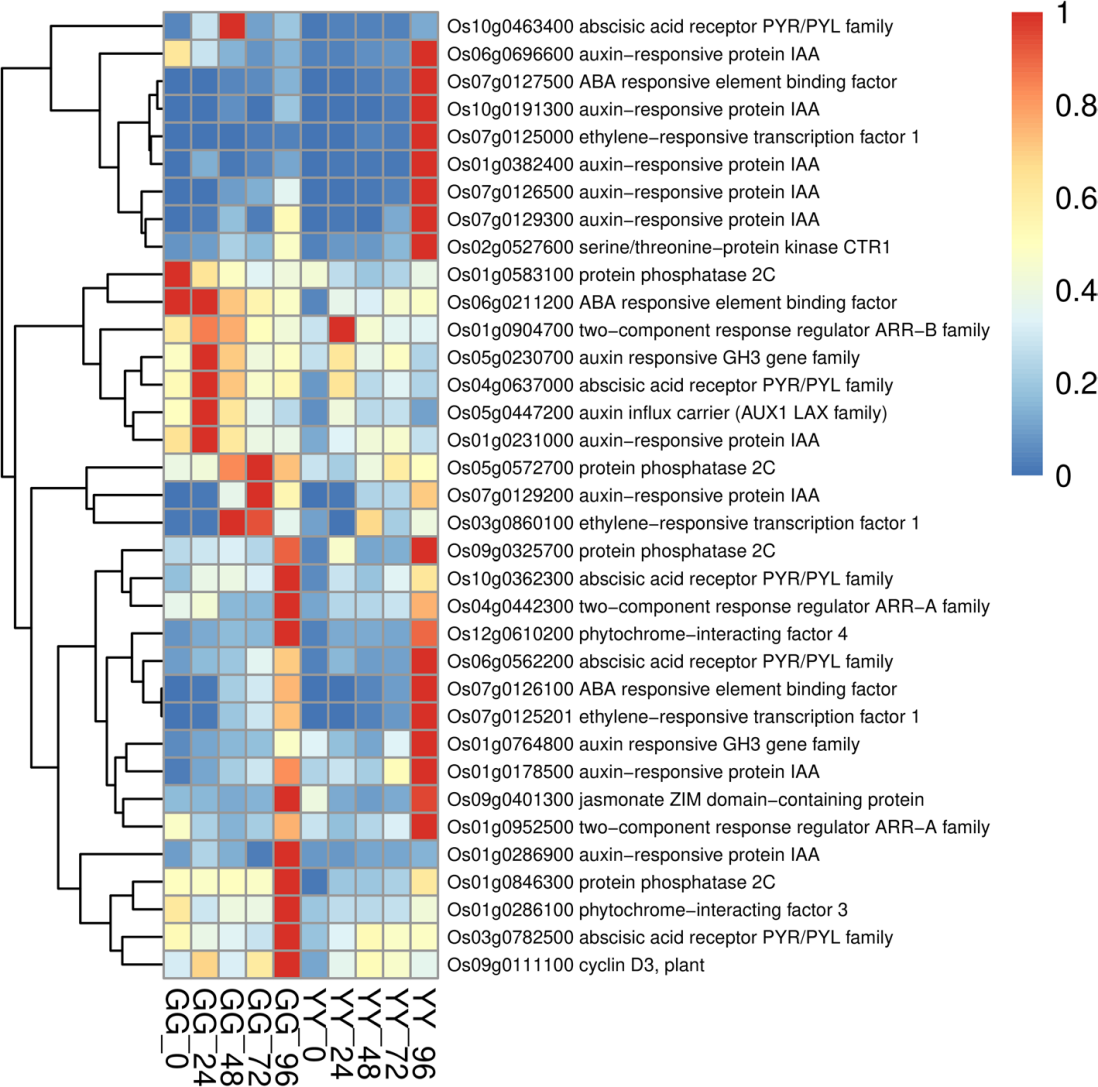
Detailed expression profiles of genes related to plant hormone signal transduction

We selected the DEGs of the two varieties at different time points for KEGG enrichment analysis (Figure S8). We found that some of the metabolic enrichments associated with resistance, namely, phenylpropanoid biosynthesis, alpha-linolenic acid metabolism, phagosomes, and the TCA cycle, were only enriched in Gangyuan8, and plant-pathogen interactions were only enriched in Yanfeng47. Based on the results of the KEGG enrichment analysis of the DEGs in Gangyuan8 at different stages, we found that immune-related metabolism was significantly enriched at the initial stage of infection, and phenylalanine metabolism was significantly enriched after 24 h and was only enriched in the resistant variety. Alpha-linolenic acid metabolism, which is highly related to jasmonic acid metabolism, was significantly enriched at 48 h. At the same time point, other resistance-related metabolisms, such as diterpenoid biosynthesis, were also significantly enriched. Based on the previous expression profile analysis, we preliminarily confirmed that the genes in clusters 9, 10 and 11 were involved in the disease resistance pathway of rice. To identify potential regulatory genes closely related to phenylalanine metabolic pathways, we identified DEGs by comparing the expression changes between the two varieties. At 96 h, the expression level of genes related to phenylalanine metabolism in the GG samples was significantly higher than that in the YY samples, and the difference between the two varieties can be seen in the heatmap (Fig. 4). Jasmonic acid is an important signaling molecule in plants, and the biosynthesis of jasmonic acid in plants originates from linolenic acid. To explore the role of the jasmonic acid pathway in rice sheath blight resistance, we drew a heatmap of the related genes involved in alpha-linolenic acid metabolism and compared the differences between the two varieties. The results showed that the expression of genes related to alpha-linolenic acid metabolism was higher in the GG samples than in the YY samples. As shown in Fig. 5, the expression level of related genes in the GG samples was always significantly higher than that in the YY samples. The expression levels of genes closely related to plant hormone signal transduction in the leaves of the two cultivars are shown in Fig. 6. From 24 to 72 h, the expression levels of related genes in the GG samples were significantly higher than those in the YY samples.

**Fig. 7:**
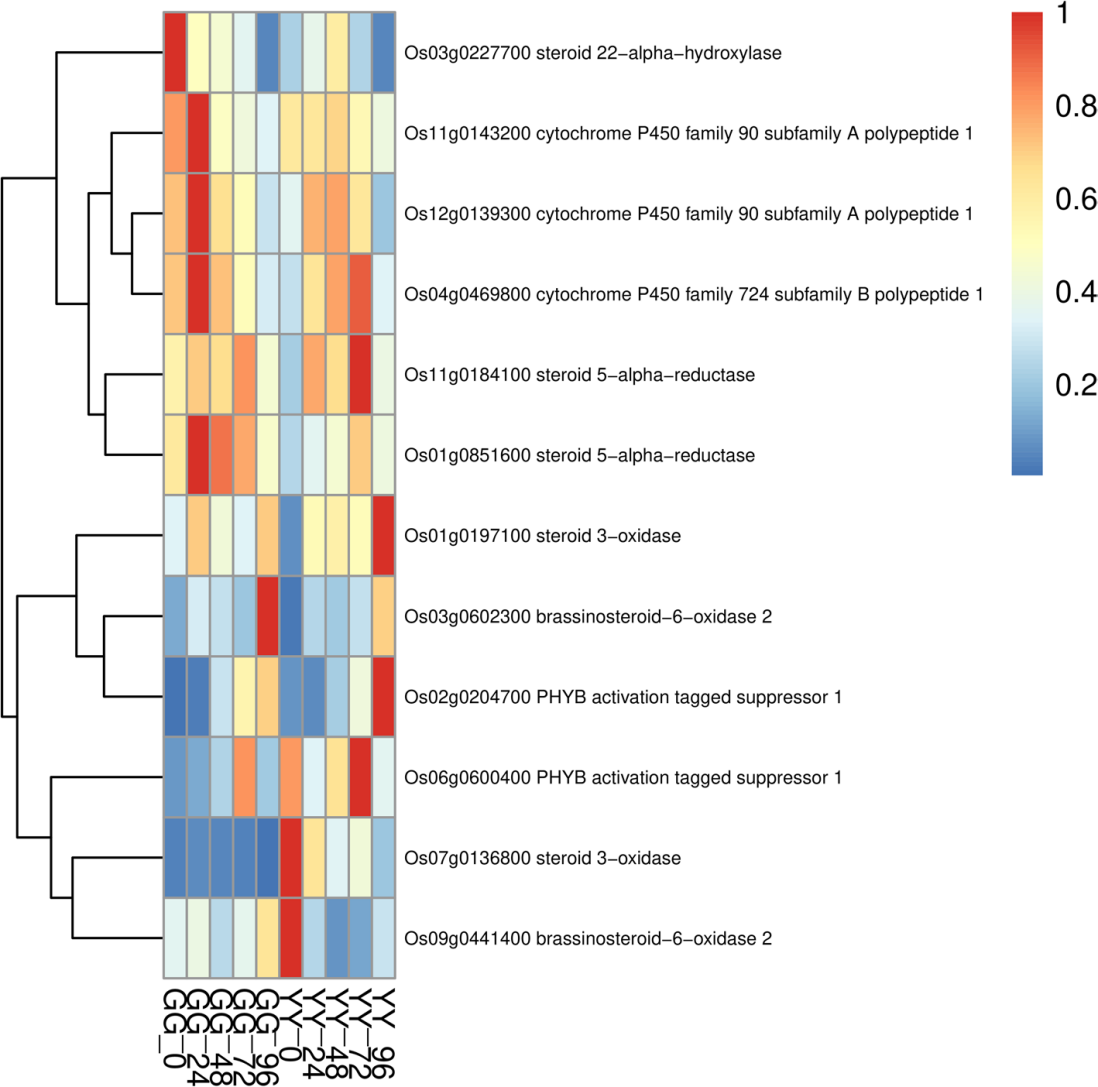
Detailed expression profiles of genes related to brassinosteroid

**Fig. 8:**
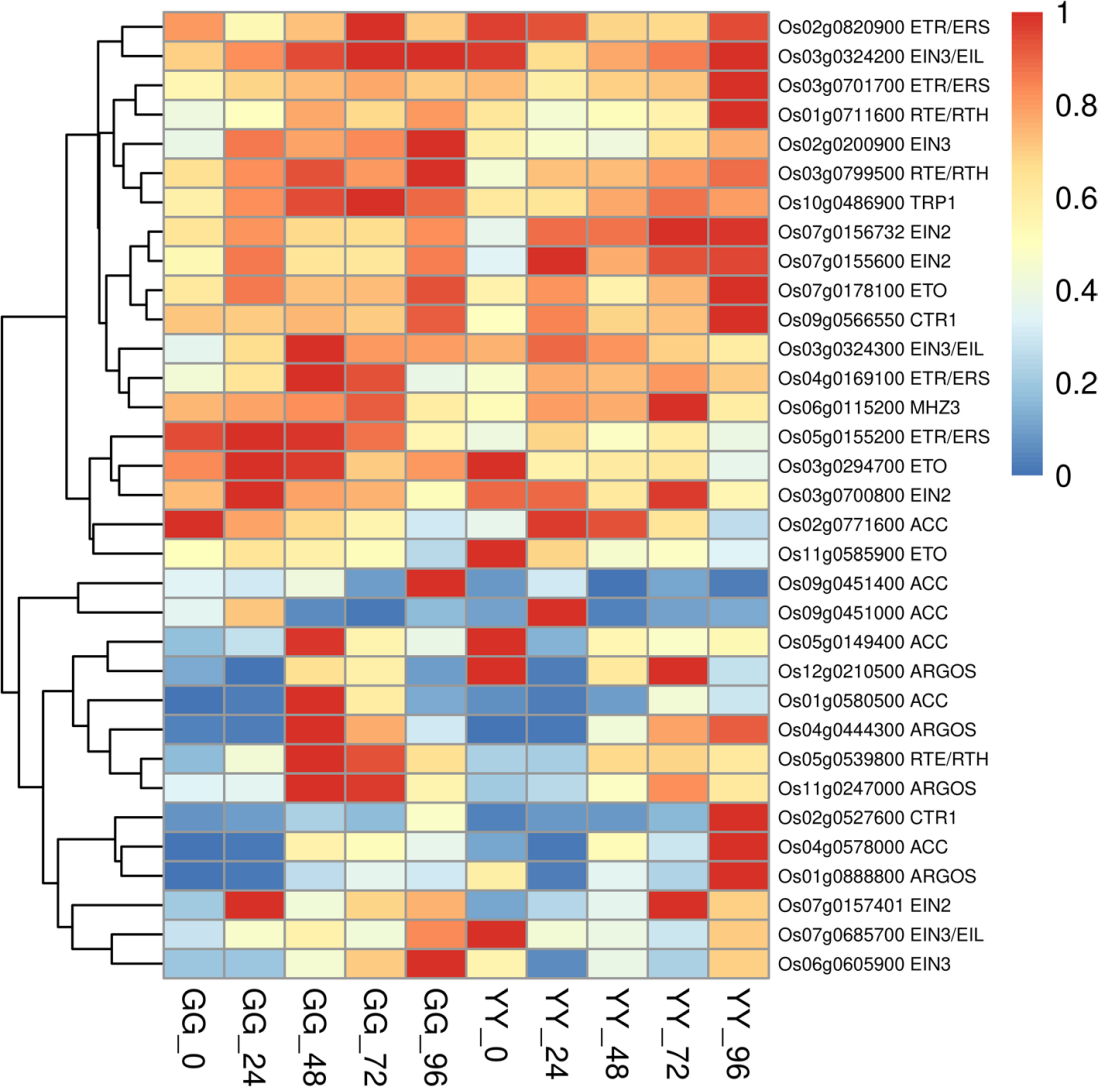
Detailed expression profiles of genes related to ethylene

Brassinosteroid (BR) is an important plant hormone, which is involved in plant growth and development. To find potential regulatory genes closely related to brassinosteroids, we determined DEGs by comparing the two cultivars. As can be seen from the heat map, *Os011G0143200* and other genes were significantly up-regulated in the resistant variety(GG) at 24 h after inoculation (Fig. 7). Ethylene (ET) is an important hormone in plants, which mainly regulates seed germination and growth, fruit ripening and plant growth. It also plays an important role in response to biological and abiotic stress. A large number of studies have shown that ethylene is involved in regulating the immune response of arabidopsis, tobacco, tomato, rice and soybean. Our results showed that in ethylene signaling pathway, more differentially expressed genes were detected in resistant and susceptible cultivars, such as *Os03g0324300* were up-regulated at the early stage, and *Os09g0451400* were up-regulated at 96 h after infection (Fig. 8).

**Fig. 9:**
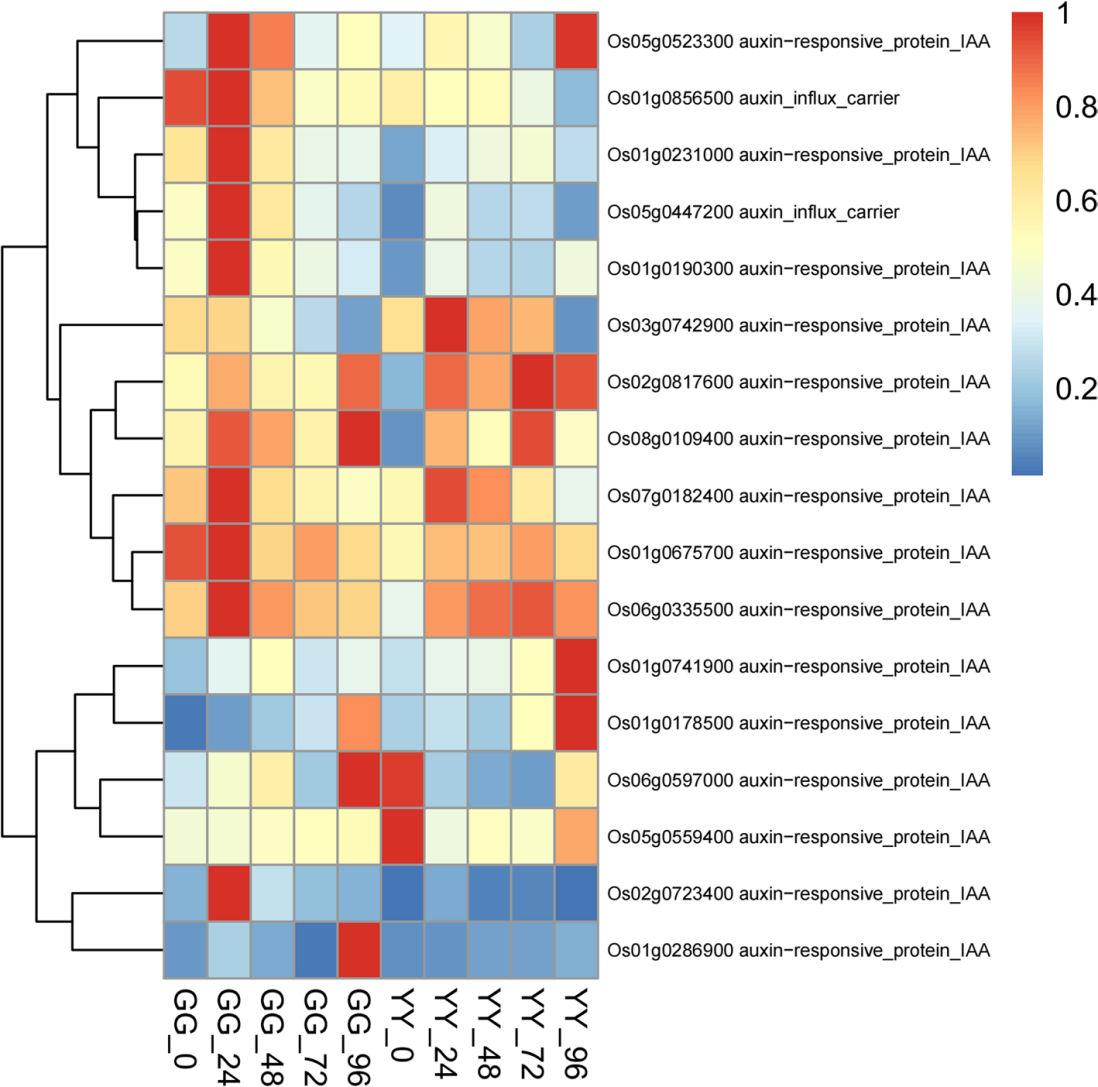
Detailed expression profiles of genes related to IAA

IAA was the first plant hormone to be discovered, which not only participated in the rice growth, but also plays an important role in immune response. Our results showed that a series of IAA responsive proteins in Gangyuan8 were up-regulated after the infection of AG1 IA, such as *Os01g0231000*, *Os01g0190300* (Fig. 9). Also some IAA-responsive genes (*Os01g0741900* and *Os02g0817600*)were highly expressed in susceptible cultivars. Therefore, whether IAA can regulate rice to defense *Rhizoctonia solani* through these genes needs to be further verified.

### Quantitative RT-PCR (qRT-PCR) validation of DEGs

To verify the reliability of the sequencing results, we selected 12 genes that were expressed in both the GG and YY samples for qPCR verification. The results showed that the expression trends of the 12 genes were similar to the sequencing results, which indicated that the sequencing results used in this study were relatively reliable (Figure S9).

## Discussion

A large number of pathogenic microorganisms have been found in the growing environment of rice and other plants and therefore threaten the normal life activities of plants [20]. To cope with this challenge, rice and other plants have developed a complex and elaborate innate immune system during their long-term coevolution with pathogens and utilize this system to recognize and resist various pathogenic microorganisms, such as fungi, bacteria, viruses, and nematodes, and to further trigger the immune defense response [28, 41]. In this study, we used RNA-seq technology to sequence the transcriptome of Gangyuan8 and Yanfeng47 leaves at different time points after AG1 IA infection and explored the resistance mechanism of rice to sheath blight by comparing the gene expression changes of materials with different resistance levels. The results showed that the two cultivars exhibited two different gene regulation patterns in response to AG1 IA infection. The results of the transcriptome analysis revealed that the related DEGs increased over time after infection. At different time points, the number of genes in Yanfeng47 was higher than that in Gangyuan8, which indicated that Yanfeng47 was under greater infection pressure.

Plant hormones such as salicylic acid, jasmonic acid and ethylene, which are important signaling molecules in the immune response of rice, have been found to extensively mediate the disease resistance response of rice [33] and help plants adapt to stress by mediating the expression of downstream genes [36]. A number of studies have shown that plant hormones are important secondary transduction signals in the rice defense response and are crucial for the activation of genes related to rice disease resistance and the transmission of downstream defense signals [1, 8, 31, 46]. Among these signals, salicylic acid and jasmonic acid are the two most important defense response hormones [2, 14, 27]. Through a series of precise positive and negative regulatory effects, these hormones can achieve a balance between growth and the stress response in rice. Jasmonic acid is a cyclopentanone derived from linolenic acid that plays a regulatory role in plant development and fungal infection [16]. In this study, genes involved in alpha-linolenic acid metabolism in Gangyuan8 leaves were significantly enriched at 24 h, which indicated that the jasmonic acid signaling pathway plays a very important role in the infection of Gangyuan8 leaves with AG1 IA. It has been speculated that jasmonic acid is involved in the immune response and related defense response of rice at the early stage of infection. In plant immune system, ethylene is thought to be cooperated with JA to induce plant resistance to necrotrophic pathogens and antagonize SA-mediated resistance to biotrophic pathogens [9]. In the process of resistance to rice blast, ethylene signaling downstream transcription factor OsEIL1 can activate OsrbohA/OsrbohB and OsOPRs expression, and then activate ROS outburst and phytoprotectin accumulation [46]. Ethephon spraying on leaves can induce and activate the expression of PRs gene. In addition, ethylene synthesis also plays an important role in rice resistance. Helliwell found that overexpression of OsACS2, a ethylene biosynthetic rate-limiting enzyme gene, significantly increased rice resistance to *M. oryzae* and *R. solani* [15]*.* OsBIHD1 binds to the promoter region of OsACO3 can activate OsACO3 and promote the expression of OsACOs in transgenic OsBIHD1 plants. These results suggested that ethylene synthesis plays an important role in OsBIHD1 positive regulating of rice disease resistance [25]. In our study, we also identified a large number of differential genes involved in ethylene metabolic pathways, their function needs to be further verified. *Os02g0527600* and *Os09g0566550* overexpressed in susceptible cultivars, they were identified with ethylene-responsive protein kinase Le-CTR1. Arabidopsis AtCTR1 is a Raf-like protein kinase that interacts with ETR1 and ERS and negatively regulates ethylene responses. So we hypothesized that the susceptibility to disease in Yanfeng47 may be related to these genes.

Brassinosteroid (BR) affect plant growth and development, cell division, cell expansion, seed germination and stem elongation. In addition, it is also an important signal involved in plant disease resistance. Studies have shown that OsSERK2 can positively regulate the immune response mediated by receptor-like kinase Xa21, can inhibit xA21-mediated resistance to Xoo in rice through reducing the expression of OsSERK2 [6]. Zuo [53] found that OsSERK1 was not involved in the defense response of rice to *M. Oryzae*. Liao [24] showed that OsSERK1 positively regulates rice resistance to *Xoo*. BSKs (Br-signaling kinase) family OSBSK1-2 is also involved in rice blast resistance, but did not participate in the response of rice to BR [43]. These results suggested that the receptor-like kinases of BR signaling pathway may positively regulate the resistance of rice. In our study, such as *Os11g0143200*、*Os12g0139300*、*Os04g0469800* and *Os01g0851600*, the expression levels of these genes increased in resistant cultivars at the early stage of infection, they were involved in BR synthesis. This result suggested that these genes may regulate BR to participate in disease resistance by affecting BR synthesis. Through functional annotations, we also found that most of these genes belonged to cytochrome P450. Cytochrome P450 were involved in the formation of various disease-resistant substances in the plant through metabolic pathways such as phytoalexin and lignin synthesis. It will be more meaningful to use mutants for phenotypic identification in the future.

Some evidence suggests that IAA can regulate rice resistance to pathogen infection [47]. Rice treated with IAA could stimulate the proliferation of Xoo and become more susceptible to disease. At the same time, Xoo infection can induce the accumulation of IAA in rice [11]. In plants, GH3 proteins catalyze the synthesis of IAA- amino acids and thus inhibit auxin. In rice, overexpression of *GH3-8*, *OsGH3-1* and *GH3-2* can reduce the content of IAA and lead to rice dwarf. However, it enhanced the resistance of rice to Xoo, *Xanthomonas oryzae* PV. *Oyrzicola* (*Xoc*) and *M. oryzae* [10, 13]. In addition, overexpression of *OsCYP71Z2* can also enhances resistance to Xoo by inhibiting IAA signal [26]. Our study showed that auxin related genes were up-regulated in susceptible and resistant cultivars, which indicated that the mechanism of auxin regulating rice disease resistance was complex.

Plant secondary metabolites play an important role in plant life and are widely involved in plant growth and development, signal transduction and pathogen defense [48, 49]. The phenylpropanoid pathway is also involved in the biosynthesis of many plant defensive compounds, including flavonoids, lignin, condensed tannins, hydroxycinnamic acid, coumarins and stilbenes [4, 37]. Arabidopsis, soybean and pepper have multiple PAL genes [3, 17]. PALs are responsive to pathogen infection and may positively regulate the accumulation of salicylic acid in maize to resist sugarcane mosaic virus (SCMV) infection [22, 52]. The KEGG results showed that phenylalanine metabolism plays an important role in the resistance of Gangyuan8 to *Rhizoctonia solani* infection, particularly at the early stage of infection. An analysis of the related genes in clusters 9, 10 and 11 showed that phenylalanine ammonia lyase genes were activated in leaves after AG1 IA inoculation. In this study, two differentially expressed PAL genes that participate in phenylalanine metabolic pathways were identified. For example, *Os04g0518400* and *Os12g0520200* are hypothesized to be involved in phenylalanine biosynthesis. However, some PAL genes (*Os08g0140300*, *Os02g0626400* and *Os05g0427400*) were highly expressed in susceptible cultivars. Gangyuan8 and Yanfeng47 were inoculated under similar environmental conditions, so how environmental conditions affect the increasing in PAL genes was unknown. It was very likely that the genotypic differences among cultivars promoted the increasing expression of PAL genes.

## Conclusions

In summary, the comparative transcriptome analysis and gene expression pattern comparison of Gangyuan8 and Yanfeng47 infected with *Rhizoctonia solani* comprehensively identified the defense mechanism involved in the response of rice to AG1 IA infection. Multiple resistance pathways were activated by AG1 IA infection, and different genes participated in the defense response and signal transduction. This result suggested that the response of rice to pathogens is regulated by multiple gene networks. Based on the comparative transcriptome analysis, a number of metabolic pathways associated with resistance were found to be significantly activated, and we focused on the biosynthesis of jasmonic acid and phenylalanine metabolism. Resistance genes belonging to the PAL gene family were identified in this study, and the upregulated expression of PAL genes indicated that these genes may play an important role in the resistance mechanism of rice to AG1 IA. Transcriptome analysis helps us better explore the molecular mechanism of rice sheath blight resistance, and the data obtained in this study can also be used to screen for candidate resistance genes that can be used for the genetic improvement of germplasm resources.

## Methods

### Plant growth and infection assay

All accession seeds used in this study were provided by Shenyang Agriculture University. The experiments were performed in Shenyang. The plots of the cultivars consisted of four-meter-long rows of 20 plants with 30-cm spacing between the rows. No fungicide was used throughout the growth period. The seeds were sown in mid-April, and the seedlings were transplanted at the end of May. Rice plants at the tillering stages were inoculated in the open field. *R. solani* strain Y-36, which was initially collected in Liaoning Province in northern China, was obtained from Shenyang Agriculture University and cultured on potato dextrose agar (PDA) plugs [19]. The cells were cultured for 7 days until the mycelia grew throughout the plate. When the plants were at the late tillering stage, the fugus plates were attached to the upper 3 leaf sheaths of rice plants and wrapped with sealing film, which is beneficial for moisturizing. They were observed every 24 h and photographed [42]. The severity of the disease was judged by measuring the area of disease spots on leaves. Photoshop software was used to convert the area of diseased spots on the photos into pixels and calculate the relative lesion area (RLA), as an evaluation index to measure the severity of disease. RLA was calculated as follows: RLA = diseased spot area/leaf area = diseased spot area pixel/leaf area pixel. Tukey method was used to analyze the RLA of different rice varieties by anova and multiple comparisons [38]. Three centimeters of the leaves were cut at five times points after inoculation (0, 24, 48, 72 and 96 h). Three biological replicates and random leaf samples were collected. The samples were rapidly frozen in liquid nitrogen and stored at -80 °C. All the samples were sent to Beijing Biomarker Technologies for transcriptome sequencing.

### RNA-seq data processing

FastQC (v0.11.9) was used to assessed the quality of the sequencing data (http://www.bioinformatics.babraham.ac.uk/projects/fastqc/). The sequences were trimmed to remove the sequencing adaptor, poly A and T tails with Trim Galore (http://www.bioinformatics.babraham.ac.uk/projects/trim_galore/) setting default values (stringency of 6 bp) and paired-end reads where kept when both pairs were longer than 40 bp. Filtered RNA-seq data was aligned to the rice genome by hisat2 (v2.2.0) software with default parameters [21]. Then the generated SAM files were converted to BAM files by Samtools software (v1.15.1) [23]. The R package HT-Seq (v0.11.2) (http://pypi.python.org/pypi/HTSeq) was applied to calculate the count of read pairs against all annotated genes. DEGs were identified using the R (v3.6.1) Bioconductor package, EdgeR (v3.28.0). Gene counts were normalized using the RLE method as imbed in EdgeR [34]. Gene expression level was quantified with FPKM (Fragments Per Kilobase per Million). DEGs were identified using a generalized linear model likelihood ratio test and Benjamini Hochberg corrected P value (FDR <  = 0.05 && abs (log2 (Treat/Control)) >  = 1).

### Screening of DEGs

The DESeq R algorithm was used for the screening of DEGs. To improve the detection rate of differentially expressed transcripts, the data were filtered to remove the transcripts with the lowest total number of 40%. The estimated size factor function was used to estimate the effective library size for the normalization of transcript counting. The dispersion was estimated using the estimated dispersion function. The nbinomTest function was used to determine whether there was differential expression between the two conditions. The false discovery rate (FDR) was controlled at 5% using the Benjamini–Hochberg method. Transcript per million (TPM) values were used to measure the proportion of transcripts in the RNA library. The overlap of differentially expressed transcripts identified from the comparison was analyzed using a Venn diagram.

### Validation of DEGs by quantitative real-time PCR (qRT-PCR)

To verify the reliability of the sequencing results of DEGs, 12 DEGs identified from Gangyuan8 and Yanfeng47 were detected by qPCR, and the specific primers are shown in Table S2. RNA Extraction was performed according to TaKaRa MiniBEST Plant RNA Extraction Kit instructions. cDNA was generated by reverse transcription using the PrimeScriptTM 1st Strand cDNA Synthesis Kit (TaKaRa, Japan). qRT-PCR experiments were analyzed using the ChamQ Universal SYBR®qPCR Master Mix real-time quantitative kit (TaKaRa) , the 2^-⊿⊿Ct^ algorithm was used to calculate the gene expression levels.

### Pattern mining based on time points and gene expression analysis

To analyze the control and treatment groups at different time points, we designed a series of formulas, including the conditional factor, the time factor and their interaction. Under this premise, we used a simplified model without interaction terms to conduct a likelihood ratio test to test whether this condition induced changes in gene transcription at any time point after the control time point (0 h after inoculation). A total of 7472 DEGs were identified between Gangyuan8 and Yanfeng47 leaves. We conducted a coexpression analysis of all tissues using the R package “pheatmap” (https://cran.r-project.org/web/packages/pheatmap/index.html). Before running the software, we calculated the relative expression values of the genes by dividing their expression level by their maximum observed FPKM value. The relative expression levels were used as input for “pheatmap” and clustered using the hierarchical clustering method.

### Functional enrichment analysis

EggNOG-mapper software (v2) was applied to annotate genes in rice (http://eggnog-mapper.embl.de/) using the protein sequences as input files. The GO and KEGG enrichment analyses of DEGs was implemented using the “clusterProfiler” R package. GO and KEGG terms with FDR <  = 0.05 were defined as enriched.

## Supplementary Information

**Additionalfile 1: Figure S1.** Phenotype identification of sheath blight diseasedetected in Gangyuan8 and Yanfeng47. **FigureS2.** The relative area of disease spot in different inoculation time point. **Figure S3.** The overall relatedness oftranscriptomes of different times. **FigureS4.** Assess the similarity between samples across conditions by PCA analyse.**Figure S5.** Functional classificationof different express genes (DEGs) in the Gangyuan8 (GG) and Yanfeng47 (YY). **Figure S6.** GO enrichment analysis ofdifferentially expressed genes. **Figure S7.**GO function enrichment analysis of clusters. **Figure S8.** KEGG enrichment analysis of differentially expressedgenes. **Figure S9.** Verification ofdifferentially expressed genes by qRT-PCR. Supplementary **Table S1.** Statistics on the number of differentially expressedgenes. Supplementary **Table S2.**Specific primers of differential gene sequences for qRT-PCR.

**Additional file 2.** Number of expressed genes in all samples (FPKM>0.5).

## Data Availability

RNA-seq reads were deposited in NCBI database under project accession PRJNA782381. All data generated or analyzed during this study are included in this article and its Additional fles. The datasets generated and analyzed during the current study are available from the corresponding author on reasonable request.

## Abbreviations

RHB: Rice sheath blight
GG: Gangyuan8
YY: Yanfeng47
*R.Solani*: *Rhizoctonia solani*
PDA: Potato dextrose agar
DEG: Differentially expressed genes
FDR: False discovery rate
TPM: Transcript per million
PCC: Pearson correlation coefficient
PCA: Principal component analysis

## References

[1] Alazem M, Lin NS (2015). Roles of plant hormones in the regulation of host-virus interactions. Mol Plant Pathol.

[2] An C, Mou Z (2011). Salicylic acid and its function in plant immunity. J Integr Plant Biol.

[3] Bednarek P, Osbourn A (2009). Plant-microbe interactions: chemical diversity in plant defense. Science.

[4] Chakraborty AK, Dustin ML, Shaw AS (2003). In silico models for cellular and molecular immunology: successes, promises and challenges. Nat Immunol.

[5] Channamallikarjuna V, Sonah H, Prasad M, Rao GJN, Chand S, Upreti HC, Singh NK, Sharma TR (2010). Identification of major quantitative trait loci qSBR11–1 for sheath blight resistance in rice. Mol Breeding.

[6] Chen X, Zuo S, Schwessinger B (2014). An XA21-associated kinase (OsSERK2) regulates immunity mediated by the XA21 and XA3 immune receptors. Mol Plant.

[7] Chen ZX, Zhang YF, Feng F, Feng MH, Jiang W, Ma YY, Pan CH, Hua HL, Li GS, Pan XB, Zuo SM (2014). Improvement of japonica rice resistance to sheath blight by pyramiding qSB-9(TQ) and qSB-7(TQ). Field Crop Res.

[8] De Vleesschauwer D, Xu J, Hofte M (2014). Making sense of hormone-mediated defense networking: from rice to Arabidopsis. Front Plant Sci.

[9] Derksen H, Rampitsch C, Daayf F (2013). Signaling cross-talk in plant disease resistance. Plant Sci.

[10] Ding X, Cao Y, Huang L, Zhao J, Xu C, Li X (2008). Activation of the indole-3-acetic acid-amido synthetase GH3-8 suppresses expansin expression and promotes salicylate-and jasmonate-independent basal immunity in rice. Plant Cell.

[11] Domingo C, Andrés F, Tharreau D, Iglesias DJ, Talón M (2009). Constitutive expression of OsGH3.1 reduces auxin content and enhances defense response and resistance to a fungal pathogen in rice. Mol Plant Microbe In.

[12] Eizenga GC, Prasad B, Jackson AK, Jia MH (2013). Identification of rice sheath blight and blast quantitative trait loci in two different O-sativa/O-nivara advanced backcross populations. Mol Breeding.

[13] Fu J, Liu H, Li Y, Yu H, Li X, Xiao J (2011). Manipulating broad-spectrum disease resistance by suppressing pathogen-induced auxin accumulation in rice. Plant Physiol.

[14] Gan L, Wu H, Wu D, Zhang Z, Guo Z, Yang N, Xia K, Zhou X, Oh K, Matsuoka M, Ng D, Zhu C (2015). Methyl jasmonate inhibits lamina joint inclination by repressing brassinosteroid biosynthesis and signaling in rice. Plant Sci.

[15] Helliwell EE, Wang Q, Yang Y (2013). Transgenic rice with inducible ethylene production exhibits broad-spectrum disease resistance to the fungal pathogens Magnaporthe oryzae and Rhizoctonia solani. Plant Biotechnol J.

[16] He Y, Zhang H, Sun Z, Li J, Hong G, Zhu Q, Zhou X, MacFarlane S, Yan F, Chen J (2017). Jasmonic acid-mediated defense suppresses brassinosteroid-mediated susceptibility to Rice black streaked dwarf virus infection in rice. New Phytol.

[17] Huang J, Gu M, Lai Z, Fan B, Shi K, Zhou YH, Yu JQ, Chen Z (2010). Functional analysis of the Arabidopsis PAL gene family in plant growth, development, and response to environmental stress. Plant Physiol.

[18] Jain M (2012). Next-generation sequencing technologies for gene expression profiling in plants. Brief Funct Genomics.

[19] Jia Y, Correa-Victoria F, McClung A, Zhu L, Liu G, Wamishe Y, Xie J, Marchetti MA, Pinson SRM, Rutger JN, Correll JC (2007). Rapid Determination of Rice Cultivar Responses to the Sheath Blight Pathogen Rhizoctonia solani Using a Micro-Chamber Screening Method. Plant Dis.

[20] Jones JDG, Dangl JL (2006). The plant immune system. Nature.

[21] Kim D, Langmead B, Salzberg SL (2015). HISAT: A fast spliced aligner with low memory requirements. Nat Methods.

[22] Kim DS, Hwang BK (2014). An important role of the pepper phenylalanine ammonia-lyase gene (PAL1) in salicylic acid-dependent signalling of the defence response to microbial pathogens. J Exp Bot.

[23] Li H, Handsaker B, Wysoker A, Fennell T, Ruan J, Homer N (2009). The Sequence Alignment/Map format and SAMtools. Bioinformatics.

[24] Liao H, Xiao X, Li X (2016). OsBAK1 is involved in rice resistance to Xanthomonas oryzae pv. oryzae PXO99. Plant Biotechnol Rep.

[25] Liu H, Dong S, Gu F, Liu W, Yang G, Huang M, Xiao W, Liu Y, Guo T, Wang H, Chen Z, Wang J (2017). NBS-LRR protein Pik-H4 interacts with OsBIHD1 to balance rice blast resistance and growth by coordinating Ethylene-Brassinosteroid pathway. Front Plant Sci.

[26] Li W, Wang F, Wang J, Jun W, Fan J, Zhu J, Yang J (2015). Overexpressing CYP71Z2 enhances resistance to bacterial blight by suppressing auxin biosynthesis in rice. PLOS ONE.

[27] Marjamaa K, Kukkola EM, Fagerstedt KV (2009). The role of xylem class III peroxidases in lignification. J EXP BOT.

[28] Molla KA, Karmakar S, Molla J, Bajaj P, Varshney RK, Datta SK, Datta K. Understanding sheath blight resistance in rice: the road behind and the road ahead. Plant Biotechnol J. 2020;18(4):895–915. 10.1111/pbi.13312 PMC7061877 31811745

[29] Noor, A.; Meier, A.; Matthew, G.; Gongora, Y.S.; Oard, J.; Jaiswal, P., Loss of premature stop codon in the Wall-Associated Kinase 91 (OsWAK91) gene confers sheath blight disease resistance in rice. *bioRxiv* 2019. 10.1101/625509. 10.3390/genes14091673 PMC10530950 37761813

[30] Okubara PA, Dickman MB, Blechl AE (2014). Molecular and genetic aspects of controlling the soilborne necrotrophic pathogens Rhizoctonia and Pythium. Plant Sci.

[31] Pieterse CM, Leon-Reyes A, Van der Ent S, Van Wees SC (2009). Networking by small-molecule hormones in plant immunity. Nat Chem Biol.

[32] Pieterse CM, Van der Does D, Zamioudis C, Leon-Reyes A, Van Wees SC (2012). Hormonal modulation of plant immunity. Annu Rev Cell Dev Biol.

[33] Robert-Seilaniantz A, Grant M, Jones JD (2011). Hormone crosstalk in plant disease and defense: more than just jasmonate-salicylate antagonism. Annu Rev Phytopathol.

[34] Robinson MD, McCarthy DJ, Smyth GK (2010). EdgeR: A Bioconductor package for differential expression analysis of digital gene expression data. Bioinformatics.

[35] Sandhya RK, Anuprita R, Santosh KS, Krishnendu C, Ramani KS (2017). Physiological basis of stagnant flooding tolerance in rice. Rice Sci.

[36] Shigenaga AM, Argueso CT (2016). No hormone to rule them all: Interactions of plant hormones during the responses of plants to pathogens. Semin Cell Dev Biol.

[37] Shine MB, Yang J, El-Habbak M, Nagyabhyru P, Fu D, Navarre D, Ghabrial S, Kachroo P, Kachroo A (2016). Cooperative functioning between phenylalanine ammonia lyase and isochorismate synthase activities contributes to salicylic acid biosynthesis in soybean. New Phytol.

[38] Shu C, Zhao M, Anderson JP, Garg G, Singh KB, Zheng W, Wang C, Yang M, Zhou E (2019). Transcriptome analysis reveals molecular mechanisms of sclerotial development in the rice sheath blight pathogen Rhizoctonia solani AG1-IA. Funct Integr Genomic.

[39] Silva J, Scheffler B, Sanabria Y, De Guzman C, Galam D, Farmer A, Woodward J, May G, Oard J (2012). Identification of candidate genes in rice for resistance to sheath blight disease by whole genome sequencing. Theor Appl Genet.

[40] Singh P, Mazumdar P, Harikrishna JA, Babu S (2019). Sheath blight of rice: a review and identification of priorities for future research. Planta.

[41] Thomma BPHJ, Nuernberger T, Joosten MHAJ (2011). Of PAMPs and Effectors: The Blurred PTI-ETI Dichotomy. Plant Cell.

[42] Venu RC, Jia Y, Gowda M, Jia MH, Jantasuriyarat C, Stahlberg E, Li H, Rhineheart A, Boddhireddy P, Singh P, Rutger JN, Kudrna D, Wing R, Nelson JC, Wang G (2007). RL-SAGE and microarray analysis of the rice transcriptome after Rhizoctonia solani infection. Mol Genet Genomics.

[43] Wang J, Shi H, Zhou L, Peng CF, Liu DY, Zhou XQ, Wu WG, Yin JJ (2017). OsBSK1-2, an orthologous of AtBSK1, is involved in rice immunity. Front Plant Sci.

[44] Wang R, Lu L, Pan X, Hu Z, Ling F, Yan Y, Liu Y, Lin Y (2015). Functional analysis of OsPGIP1 in rice sheath blight resistance. Plant Mol Biol.

[45] Xue X, Cao ZX, Zhang XT, Wang Y, Zhang YF, Chen ZX, Pan XB, Zuo SM (2016). Overexpression of OsOSM1 enhances resistance to rice sheath blight. Plant Dis.

[46] Yang C, Li W, Cao J (2017). Activation of ethylene signaling pathways enhances disease resistance by regulating ROS and phytoalexin production in rice. Plant J.

[47] Yang DL, Yang Y, He Z (2013). Roles of plant hormones and their interplay in rice immunity. Mol Plant.

[48] Yuan W, Jiang T, Du K, Chen H, Cao Y, Xie J, Li M, Carr JP, Wu B, Fan Z, Zhou T (2019). Maize phenylalanine ammonia-lyases contribute to resistance to Sugarcane mosaic virus infection, most likely through positive regulation of salicylic acid accumulation. Mol Plant Pathol.

[49] Yu O, Jez JM. Nature’s assembly line: biosynthesis of simple phenylpropanoids and polyketides. Plant J. 2008;54(4):750–62. 10.1111/j.1365-313X.2008.03436.x 18476876

[50] Zakiah RM, Md AA, Md SA, Md AR, Md RB, Md SM, Khandakar MI, Md AL, Mohammad AIK (2016). Morphological and Genetical Variability among Rhizoctonia solani Isolates Causing Sheath Blight Disease of Rice. Rice Sci.

[51] Zhang J, Chen L, Fu C, Wang L, Liu H, Cheng Y, Li S, Deng Q, Wang S, Zhu J, Liang Y, Li P, Zheng A (2017). Comparative Transcriptome Analyses of Gene Expression Changes Triggered by Rhizoctonia solani AG1 IA Infection in Resistant and Susceptible Rice Varieties. Front Plant Sci.

[52] Zheng A, Lin R, Zhang D, Qin P, Xu L, Ai P, Ding L, Wang Y, Chen Y, Liu Y, Sun Z, Feng H, Liang X, Fu R, Tang C, Li Q, Zhang J, Xie Z, Deng Q, Li S, Wang S, Zhu J, Wang L, Liu H, Li P (2013). The evolution and pathogenic mechanisms of the rice sheath blight pathogen. Nat Commun.

[53] Zuo S, Zhou X, Chen M (2014). OsSERK1 regulates rice development but not immunity to Xanthomonas oryzae pv. oryzae or Magnaporthe oryzae. J Integr Plant Biol.

[54] Zuo SM, Zhu YJ, Yin YJ, Wang H, Zhang YF, Chen ZX, Gu SL, Pan XB (2014). Comparison and confirmation of quantitative trait loci conferring partial resistance to rice sheath blight on chromosome 9. Plant Dis.

[55] Zuo S, Yin Y, Zhang L, Zhang Y, Chen Z, Gu S, Zhu L, Pan X (2011). Effect and breeding potential of qSB-11(LE), a sheath blight resistance quantitative trait loci from a susceptible rice cultivar. Can J Plant Sci.

