# Supplementary material for: Comparative transcriptome analysis of rice cultivars resistant and susceptible to Rhizoctonia solani AG1-IA
Source: BMC Genomics. 2022 Aug 19;23:606. doi: 10.1186/s12864-022-08816-x (PMC9392349; doi:10.1186/s12864-022-08816-x)
Supplement: Supplementary file 1 — Additionalfile 1: Figure S1. Phenotype identification of sheath blight diseasedetected in Gangyuan8 and Yanfeng47. FigureS2. The relative area of disease spot in different inoculation time point. Figure S3. The overall relatedness oftranscriptomes of different times. FigureS4. Assess the similarity between samples across conditions by PCA analyse.Figure S5. Functional classificationof different express genes (DEGs) in the Gangyuan8 (GG) and Yanfeng47 (YY). Figure S6. GO enrichment analysis ofdifferentially expressed genes. Figure S7.GO function enrichment analysis of clusters. Figure S8. KEGG enrichment analysis of differentially expressedgenes. Figure S9. Verification ofdifferentially expressed genes by qRT-PCR. Supplementary Table S1. Statistics on the number of differentially expressedgenes. Supplementary Table S2.Specific primers of differential gene sequences for qRT-PCR. [file 12864_2022_8816_MOESM1_ESM.zip › FigureS8.pdf]

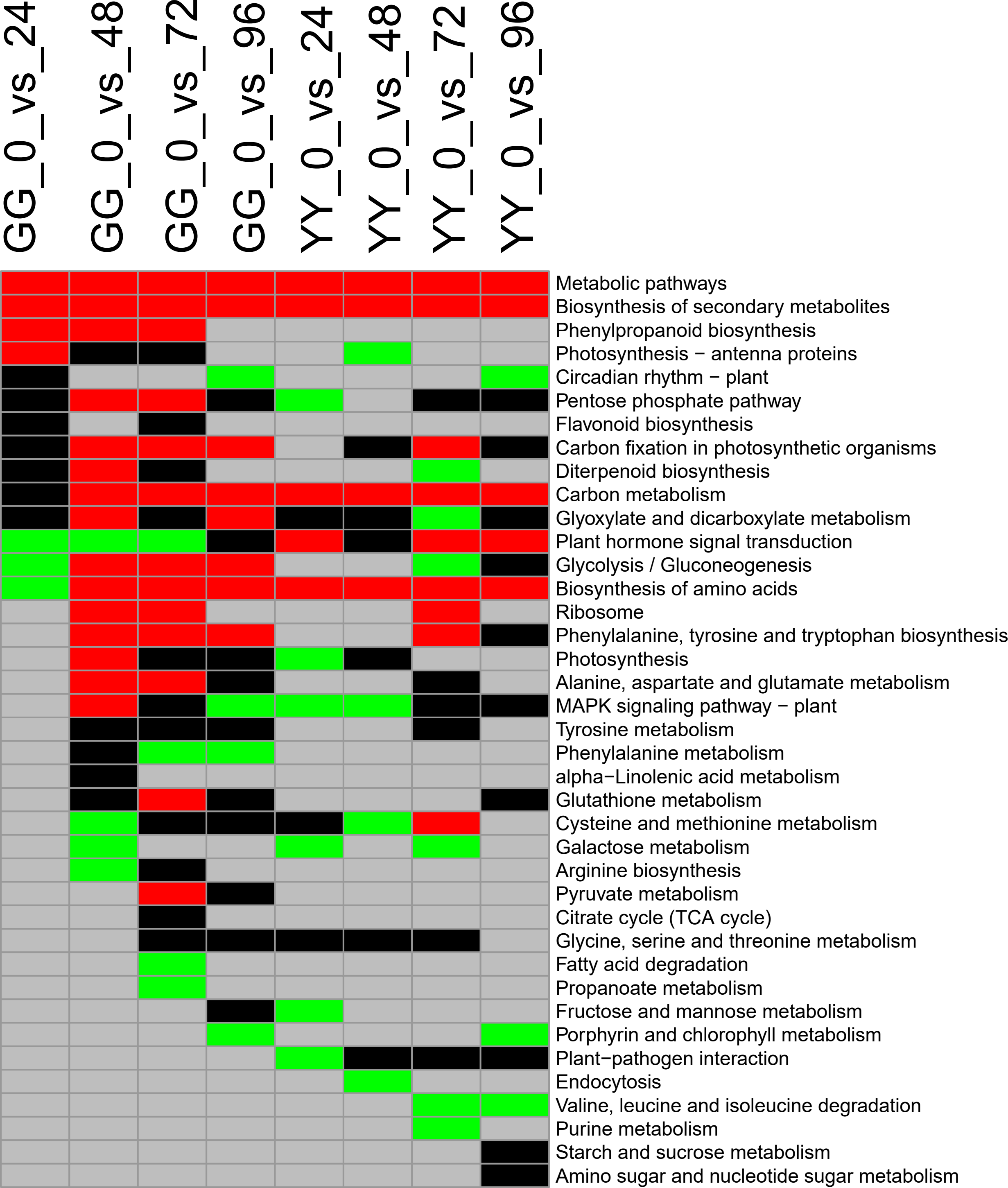
GG_0_vs_24
GG_0_vs_48
GG_0_vs_72
GG_0_vs_96
YY_0_vs_24
YY_0_vs_48
YY_0_vs_72
YY_0_vs_96
Metabolic pathways
Biosynthesis of secondary metabolites
Phenylpropanoid biosynthesis
Photosynthesis − antenna proteins
Circadian rhythm − plant
Pentose phosphate pathway
Flavonoid biosynthesis
Carbon fixation in photosynthetic organisms
Diterpenoid biosynthesis
Carbon metabolism
Glyoxylate and dicarboxylate metabolism
Plant hormone signal transduction
Glycolysis / Gluconeogenesis
Biosynthesis of amino acids
Ribosome
Phenylalanine, tyrosine and tryptophan biosynthesis
Photosynthesis
Alanine, aspartate and glutamate metabolism
MAPK signaling pathway − plant
Tyrosine metabolism
Phenylalanine metabolism
alpha−Linolenic acid metabolism
Glutathione metabolism
Cysteine and methionine metabolism
Galactose metabolism
Arginine biosynthesis
Pyruvate metabolism
Citrate cycle (TCA cycle)
Glycine, serine and threonine metabolism
Fatty acid degradation
Propanoate metabolism
Fructose and mannose metabolism
Porphyrin and chlorophyll metabolism
Plant−pathogen interaction
Endocytosis
Valine, leucine and isoleucine degradation
Purine metabolism
Starch and sucrose metabolism
Amino sugar and nucleotide sugar metabolism
