# Supplementary material for: Comparative transcriptome analysis of rice cultivars resistant and susceptible to Rhizoctonia solani AG1-IA
Source: BMC Genomics. 2022 Aug 19;23:606. doi: 10.1186/s12864-022-08816-x (PMC9392349; doi:10.1186/s12864-022-08816-x)
Supplement: Supplementary file 1 — Additionalfile 1: Figure S1. Phenotype identification of sheath blight diseasedetected in Gangyuan8 and Yanfeng47. FigureS2. The relative area of disease spot in different inoculation time point. Figure S3. The overall relatedness oftranscriptomes of different times. FigureS4. Assess the similarity between samples across conditions by PCA analyse.Figure S5. Functional classificationof different express genes (DEGs) in the Gangyuan8 (GG) and Yanfeng47 (YY). Figure S6. GO enrichment analysis ofdifferentially expressed genes. Figure S7.GO function enrichment analysis of clusters. Figure S8. KEGG enrichment analysis of differentially expressedgenes. Figure S9. Verification ofdifferentially expressed genes by qRT-PCR. Supplementary Table S1. Statistics on the number of differentially expressedgenes. Supplementary Table S2.Specific primers of differential gene sequences for qRT-PCR. [file 12864_2022_8816_MOESM1_ESM.zip › Table S2.docx]

Supplementary Table 2. Specific primers of differential gene sequences for qRT-PCR

|  | **Gene** | **Forward primers** | **Reverse primers** |
| --- | --- | --- | --- |
| 1 | Os05g0102000 | TCCACTTCCTGTCTAGGGCA | CCTGGAACTGAGCCCTGTAG |
| 2 | Os04g0358000 | TTGAGGTTGGCTCTGCGATT | GCCTCCTGCTTTCCAGAGGT |
| 3 | Os09g0503100 | CCAGAAGGCCTCAAGCATGT | TGAATCTTCCAGTCGATGGGG |
| 4 | Os01g0583100 | ATCCCGCTATGGATGCTCAC | GACAGTAATTTGCAACCTGCG |
| 5 | Os05g0572700 | GCTGGAGGCAGAGTCATCAA | CCTGAAATTCGAGATGCCTATGG |
| 6 | Os09g0438000 | CTGTCCATGGTGCAAGCTCT | TGCAAAATGTGTCCTCACCCTT |
| 7 | Os05g0230700 | CATTGGCTTCACAACTGGCAA | TATCAACTGCCCTTGGAGCAA |
| 8 | Os04g0637000 | GTTCGTTACCCAGGCAGATCA | GGAAGCGCTGGAAGTAGTCC |
| 9 | Os05g0447200 | GATCGCCTGTGCAAGTAACAT | GTGTAGGTGGTCATGCCGAG |
| 10 | Os01g0231000 | CGGTAAGGAAGGTGCTGAGAA | CAAGAGTCGGTGAACATCTCCC |
| 11 | Os10g0362300 | AGGCGGTGCGATTTGATGTC | GATACAAGTACAGCCAACTCTATGC |
| 12 | Os05g0572700 | GCTGGAGGCAGAGTCATCAA | CCTGAAATTCGAGATGCCTATGG |
